# Myopericytoma Masquerading as Dupuytren’s Disease: A Case Report and Systematic Literature Review

**DOI:** 10.3390/jcm14113703

**Published:** 2025-05-25

**Authors:** Gianluca Marcaccini, Ishith Seth, Jennifer Novo, Marcus Bautista, Lakal Ruhunage, Saiuj Bhat, Roberto Cuomo, Warren M. Rozen

**Affiliations:** 1 Department of Plastic and Reconstructive Surgery, University of Siena, 53100 Siena, Italy; robertocuomo@outlook.com; 2 Faculty of Medicine and Surgery, Peninsula Clinical School, Monash University, Clayton, VIC 3199, Australia; 3 Department of Plastic and Reconstructive Surgery, Peninsula Health, Frankston, Melbourne, VIC 3199, Australia; marcus.bautista92@gmail.com (M.B.); lakalruhunage@gmail.com (L.R.); warrenrozen@hotmail.com (W.M.R.); 4 Faculty of Medicine and Surgery, Notre Dame University, Notre Dame, NSW 2002, Australia; jenny.novo@gmail.com; 5 Department of Orthopaedic Surgery, Royal Perth Hospital, Perth, WA 6000, Australia; saiuj.bhat@gmail.com

**Keywords:** myopericytoma, Dupuytren’s disease, vascular tumour, fibroinflammatory pathways, histopathology, case report, systematic review

## Abstract

**Background:** Myopericytoma is a rare benign vascular tumour characterised by concentric spindle cell proliferation around blood vessels, often misdiagnosed due to its resemblance to other soft tissue masses. Dupuytren’s disease (DD), a fibroproliferative disorder of the palmar fascia, causes progressive contractures, typically affecting the ring and little fingers. While these conditions are well-documented individually, their coexistence in the same region is rare and diagnostically challenging. **Case Presentation:** This report highlights a 67-year-old male with longstanding DD and a recurrent palmar mass initially attributed to fibrosis. Magnetic resonance imaging revealed hallmark vascular features suggestive of myopericytoma, confirmed by histopathological analysis showing spindle cell proliferation and immunohistochemical positivity for alpha-smooth muscle actin and h-caldesmon. Concurrent DD, characterised by fibrosis and activated myofibroblasts, further complicated the clinical picture. **Methodology:** PubMed, Scopus, Web of Science, and Embase databases were searched from January 1901 to December 2024, and 20 studies were found, reporting 41 cases of myopericytoma in hand and upper extremity. Histopathological analysis consistently showed spindle cell proliferation and smooth muscle actin positivity. Coexistence with DD was rare, highlighting the need for detailed imaging and histological evaluation for accurate diagnosis. **Conclusions:** This case emphasises the complexity of differentiating overlapping pathologies. Surgical excision of myopericytoma and tailored DD management yielded favourable outcomes. Further research into shared fibroinflammatory pathways, including tumour necrosis factor-alpha and interleukin-6, may enhance diagnostic accuracy and treatment strategies for overlapping conditions.

## 1. Introduction

Myopericytoma is a rare benign tumour that arises from perivascular myoid cells, displaying a characteristic concentric arrangement of spindle cells around blood vessels. It is predominantly found in subcutaneous tissues of the extremities and is more common in young male adults. Myopericytoma typically presents as a well-defined, slow-growing, painless mass, although atypical presentations, including painful and rapidly growing lesions, have been documented. The tumour is generally indolent, with a low risk of malignancy or recurrence following complete surgical excision [1,2]. Histopathologically, myopericytoma is distinguished by its concentric perivascular proliferation of spindle-shaped myoid cells and immunohistochemical positivity for alpha-smooth muscle actin and h-caldesmon. These features are pivotal in differentiating it from other vascular or soft tissue neoplasms, such as angioleiomyomas, glomus tumours, and sarcomas [3].

Dupuytren’s disease (DD) is a fibroproliferative disorder of the palmar fascia, leading to progressive thickening and shortening of fibrous cords and resulting in flexion contractures of the fingers. This condition predominantly affects the ring and little fingers, with a higher prevalence in older male populations of European descent [4]. DD’s pathogenesis is linked to myofibroblasts’ activation, driven by inflammatory cytokines such as tumour necrosis factor-alpha and interleukin-6 within a fibrotic microenvironment. Chronic inflammation and myofibroblast activation perpetuate the contracture process, underscoring the disease’s complex and multifactorial nature [5].

While both conditions are independently well-characterised, the coexistence of myopericytoma and DD in the same anatomical region is exceedingly rare, presenting unique diagnostic and therapeutic challenges. Both diseases share overlapping features, including chronic inflammation, myofibroblast activation, and involvement of inflammatory cytokines, suggesting potential intersections in their pathophysiology. For instance, the perivascular localisation and myoid differentiation in myopericytoma may share parallels with the fibroinflammatory pathways implicated in DD. Shared cytokine profiles, such as elevated tumour necrosis factor-alpha and interleukin-6, further underscore possible molecular overlaps [5,6].

The clinical overlap between these conditions can complicate diagnosis. Myopericytoma often mimics benign or malignant tumours, including ganglion cysts, glomus tumours, and sarcomas, while DD may present as painless nodules or fibrous cords in the palm. This clinical similarity underscores the importance of advanced diagnostic techniques. Magnetic resonance imaging can help differentiate myopericytoma by identifying hallmark vascular features, such as intense post-contrast enhancement and a central vascular pedicle, which are not typically observed in DD [2,7,8]. Histopathological and immunohistochemical analyses remain crucial for confirming the diagnosis [9].

**Figure 1:**
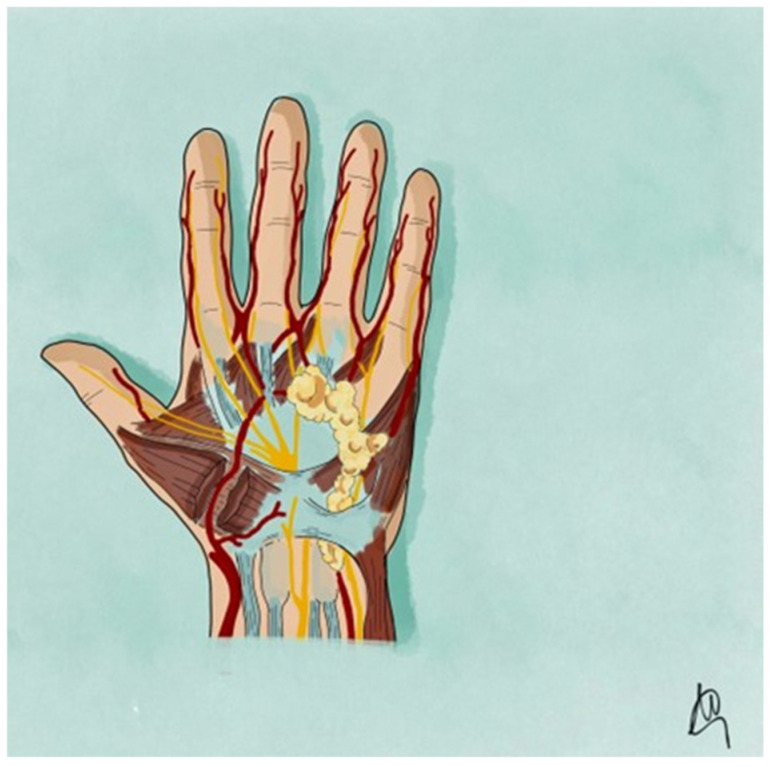
Schematic diagram of myopericytoma arising from the volar aspect of the hand.

This report describes a case of a 67-year-old male with longstanding DD who developed a coexisting myopericytoma in the palmar region of the hand. The rarity of this coexistence provides an opportunity to explore potential shared pathophysiological mechanisms and discuss implications for diagnosis and management. To the best of our knowledge, no previous publication has documented a histologically confirmed myopericytoma arising within tissue affected by established Dupuytren’s disease. The present report therefore represents the first such case in the literature, underscoring the need for heightened diagnostic vigilance when evaluating atypical or painful palmar nodules. To enhance anatomical consistency and clinical relevance, our systematic review specifically focused on cases of myopericytoma involving the hand and upper extremity. Lesions arising in other anatomical regions (e.g., lower limb, head/neck, trunk) were excluded from pooled statistical analysis (Figure 1).

## 2. Materials and Methods

### 2.1. Search Strategy

This study followed PRISMA guidelines for conducting a systematic literature review. Databases searched included PubMed, Scopus, Web of Science, and Embase from January 1901 to December 2024. Search terms incorporated combinations of keywords and Boolean operators such as (“Myopericytoma” OR “soft tissue tumour”) AND (“Hand” OR “Dupuytren” OR “Dupuytren’s Disease”). The search included articles published in English involving human subjects of any age or gender. No time restrictions were applied. This review follows the PRISMA guidelines but was not registered.

### 2.2. Literature Search

**Figure 2:**
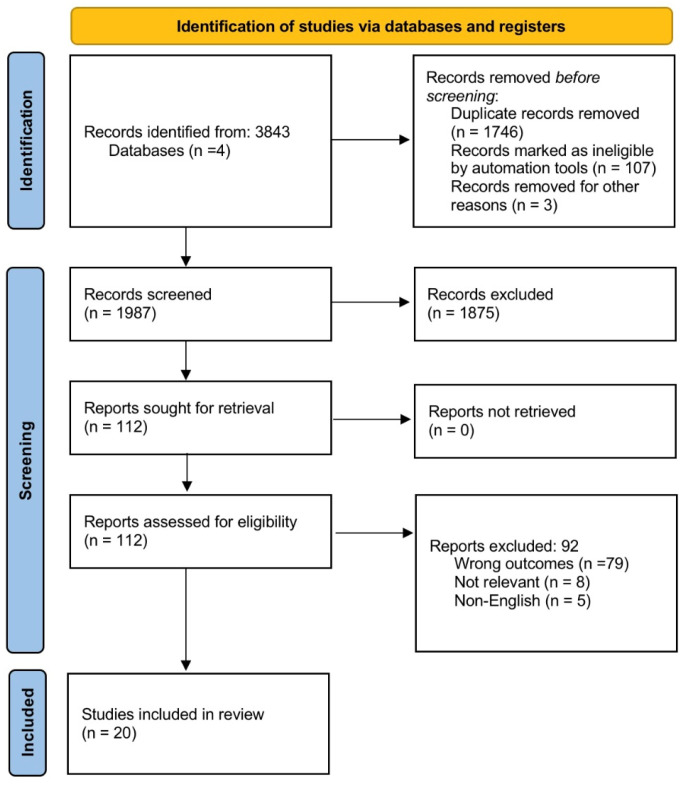
PRISMA diagram of study selection.

The initial search yielded 3843 studies. Duplicate records were removed, leaving 1987 studies for title and abstract screening. Two independent reviewers manually screened all titles and abstracts for relevance and eligibility; no automation tools were used during the selection process. A total of 112 full-text articles were assessed for eligibility, with 20 meeting the inclusion criteria. Exclusion criteria included reviews, letters to the editor, editorials, non-original studies, non-English articles, and cases lacking sufficient clinical or histopathological detail for qualitative analysis (Figure 2).

### 2.3. Data Extraction

A structured data extraction template was used to collect key information from each included study. Data points included author details, year of publication, patient demographics, clinical presentation, lesion characteristics (size, location, number), histopathological findings, immunohistochemical markers, treatment, and recurrence. Discrepancies in data extraction were resolved by consensus or consultation with a third reviewer.

To ensure anatomical consistency, we restricted the pooled statistical analysis to cases involving the upper extremity (hand, fingers, forearm, and upper arm). Tumours located in the lower limbs, trunk, or head/neck were excluded from quantitative synthesis. A sensitivity analysis was performed on the two largest series [10,11] to isolate upper-limb cases and assess for anatomical bias in demographic or outcome trends (Table 1).

### 2.4. Quality Assessment

The methodological quality of the included studies was assessed using a tool proposed by Murad et al., which evaluates four domains: selection, ascertainment, causality, and reporting, based on eight questions. The results are reported in Table 2.

### 2.5. Case Description

A 67-year-old male presented with a 12-month history of a painful, stinging sensation and callous formation over the palmar region of his left fourth digit. The patient had a 30-year history of DD involving the same digit, previously managed with partial fasciectomy. On physical examination, a large, firm nodule with significant contracture was observed in the left ring finger, consistent with recurrent DD. The patient’s medical history included chronic myeloid leukaemia (CML), chronic kidney disease (CKD), fatty liver disease, osteoarthritis (OA), and gastro-oesophageal reflux disease (GORD). A family history of DD was also reported, but no occupational risk factors were identified, as the patient was a retired financial planner.

**Figure 3:**
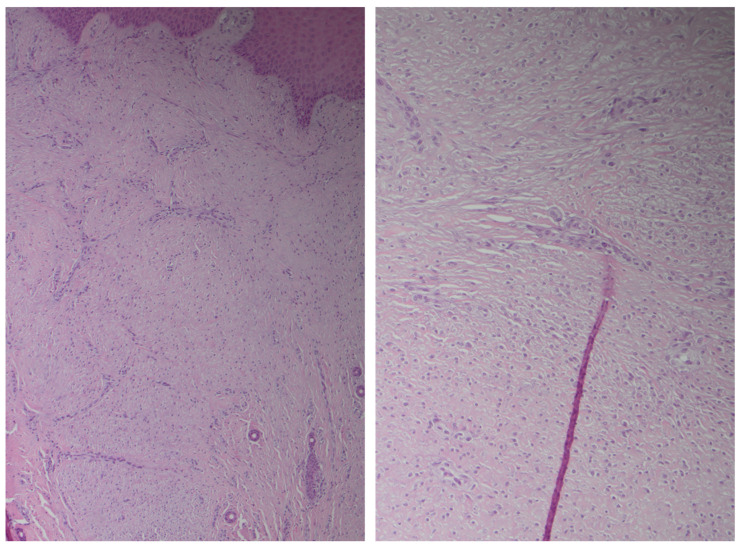
Intermediate and high power views of bland ovoid cells with compressed blood vessels interspersed.

**Figure 4:**
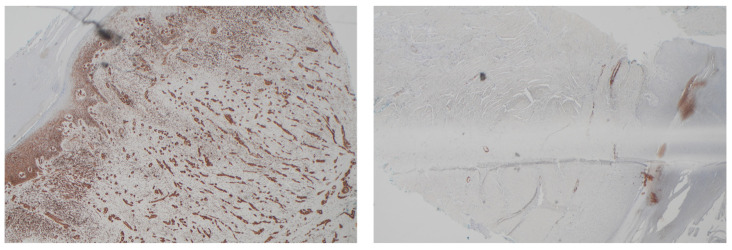
Diffuse staining with SMA and caldesmon and negative staining for desmin.

**Figure 5:**
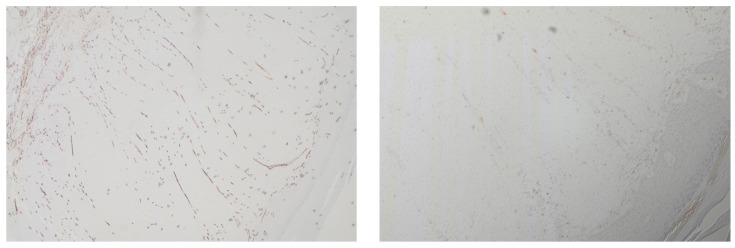
Background vessel staining with CD34, negative in lesional cells and negative staining for CD68.

**Figure 6:**
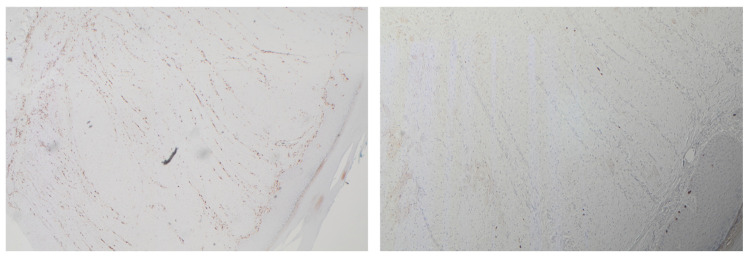
Background staining of Factor XIIIa, negative in lesion.

Clinical suspicion of a coexisting soft tissue mass prompted further investigation. Ultrasound revealed a well-circumscribed, hypoechoic lesion with increased vascularity. MRI demonstrated a 2 × 1.5 cm subcutaneous mass with intense post-contrast enhancement, T1 isointensity to muscle, and T2 hyperintensity, consistent with myopericytoma. Surgical excision of the mass was performed, with histopathology confirming the diagnosis of myopericytoma. The patient tolerated the procedure well, and subsequent follow-up showed no recurrence at 6 months of myopericytoma or worsening of DD symptoms (Figure 3, Figure 4, Figure 5 and Figure 6).

No concurrent partial fasciectomy or cord excision was performed at the index operation. The patient had undergone a limited fasciectomy 12 years earlier and, at presentation, demonstrated only a mild residual contracture (Tubiana stage I) that was asymptomatic. Because the chief complaint was pain arising from the palmar mass, the surgical plan was limited to en-bloc excision of the tumour. The option of staged surgery for recurrent Dupuytren contracture was discussed pre-operatively, and the patient elected for surveillance with the understanding that further intervention will be considered should functional impairment progress.

## 3. Results

We identified 41 myopericytomas arising in the hand or upper extremity: 16 from Mentzel, 5 from Dray and 18 additional reports encompassing 20 tumours (17 single-case reports plus one 3-case series). Gender was documented for 17 tumours and showed a male predominance (11/17, 65%). Patient age ranged from 13 to 87 years (median 52 years). Tumours were solitary in 36/41 (88%) cases; multifocal nodules were described in the Mentzel series and in one triple-digit case report. Two-thirds of lesions were painless at presentation, whereas one-third produced pain—usually from nerve compression or recent trauma. Tumour size varied from 5 mm to 90 mm (median 24 mm).

All cases exhibited the diagnostic concentric perivascular proliferation of bland spindle-to-ovoid myoid cells. Immunohistochemistry was uniformly positive for smooth-muscle actin (40/40, 100%; one case lacked immunostaining data) and variably positive for h-caldesmon (≈70%), desmin (≈45%) and vimentin (≈30%); S-100, CD34 and CD31 were consistently negative when tested.

Follow-up data were available for 37 tumours (median 2 years; range 81 days–9 years). Only one malignant recurrence occurred—within the Mentzel series (1/16, 6%), corresponding to 1/41 (2.4%) of the upper-limb cohort. All other lesions, including those up to 90 mm, remained disease-free after complete excision. No case arose in tissue affected by pre-existing Dupuytren’s disease, and no consistent clinical or environmental risk factor was discernible.

### Questions

1: Patient represents whole experience of investigator.

2: Exposure adequately ascertained

3: Outcome adequately ascertained

4: Alternate causes ruled out

5: Challenge/rechallenge phenomenon (not used)

6: Dose-response challenge (not used)

7: Adequate follow-up

8: Replicable

## 4. Discussion

Our synthesis of the 41 upper-extremity myopericytomas portrays a tumour that is uncommon yet remarkably consistent in behaviour. The cases range widely in age (13–87 years; median 52) but show a clear male skew (≈65% of those with known sex) and almost always present as a single lesion (88%), most frequently painless unless nerve irritation or a recent injury intervenes. Sizes extend from tiny nodules to 9 cm (median 24 mm), yet all share an unmistakable histological “onion-skin” pattern of bland spindle-to-ovoid myoid cells around vascular channels. This morphology is mirrored immunohistochemically by uniform SMA reactivity, frequent h-caldesmon and desmin co-expression, variable vimentin positivity, and consistent absence of S-100, CD34 and CD31, thereby excluding schwannian, solitary-fibrous and endothelial mimics. Surgical excision is typically decisive: across 37 lesions followed for up to nine years, only one malignant recurrence (2.4%) was documented, and even the largest tumours remained disease-free after complete removal. Finally, the review found no example arising within Dupuytren-affected fascia, suggesting that Dupuytren’s disease is not a predisposing condition; nevertheless, the cytokine-rich, myofibroblast-laden milieu of palmar fibromatosis could still foster perivascular myoid proliferation and warrants targeted molecular study

The pronounced predilection for the hands and fingers (65%) carries important diagnostic implications, as these tumors can mimic more common hand pathologies such as ganglion cysts, giant cell tumors of the tendon sheath, or glomus tumors [2,22]. Additionally, overlap with Dupuytren’s disease can obscure diagnosis, particularly in patients who already have palmar nodules or fibrotic contractures. While myopericytomas can be painless in the majority of cases, pain, seen here in 32%, often arises from nerve involvement and underscores the relevance of high-resolution imaging for proper lesion characterization. Advanced MRI typically reveals intense contrast enhancement with prominent vascular channels and a perivascular or “onion-skin” arrangement, findings not typically associated with the fibroproliferative bands seen in DD [2]. Such detailed imaging, correlated with thorough clinical evaluation, remains paramount for distinguishing myopericytoma from benign fibrous or vascular entities, as well as from rare malignant sarcomas. Although Dupuytren’s disease typically manifests as painless palmar nodules that can be diagnosed clinically, the abrupt onset of pain, rapid enlargement, or neuro-vascular symptoms should raise concern for an alternative pathology. High-resolution ultrasound is an appropriate first-line investigation in this scenario; MRI should be reserved for lesions that remain indeterminate on sonography or when a vascular or neoplastic mass is suspected, as it offers superior soft-tissue contrast and characteristic flow-void depiction [2,10,22]. Consequently, a painful nodule in established DD does not mandate routine MRI; rather, imaging should be tailored to the clinical context.

Histopathological confirmation hinges on recognizing the distinctive concentric perivascular arrangement of spindle-to-ovoid cells, which may otherwise be misinterpreted as angioleiomyoma, glomus tumor, or epithelioid sarcoma if one relies solely on gross histologic patterns [3,14]. Immunohistochemistry remains the gold-standard adjunct, with 100% SMA positivity highlighting smooth muscle–like differentiation. Variable yet frequent expression of h-caldesmon and desmin further supports the myoid lineage, whereas negative staining for markers such as S100, CD34, and CD31 rules out schwannomas, solitary fibrous tumors, and vascular endothelial lesions, respectively. In the reviewed literature, accurate diagnosis through this immunophenotypic profile consistently guided surgical management, which proved curative in the overwhelming majority of cases. Even in instances where lesions were large or anatomically complex, complete excision resulted in sustained local control, reflecting the low propensity of myopericytomas for recurrence and malignant transformation.

Several additional findings add to our understanding of the fibroinflammatory and vascular underpinnings of these tumors. A growing body of work suggests that myopericytomas may share certain inflammatory or myofibroblastic pathways seen in conditions like DD, given the mutual expression of alpha-smooth muscle actin and involvement of inflammatory cytokines (TNF-α, IL-6). Although a direct causal link remains hypothetical, this overlap raises intriguing questions about whether chronic inflammation and vascular alterations might predispose certain patients to fibroproliferative changes in the hand, potentially explaining the rare coexistence of myopericytoma and DD [19]. Advanced imaging, molecular profiling, and case documentation could help delineate these mechanisms more precisely and foster targeted interventions, such as anti-fibrotic agents or therapies modulating hypoxia-inducible factors (HIFs) and vascular endothelial growth factor (VEGF).

To our knowledge, this represents the most extensive synthesis specifically addressing myopericytomas of the hand and upper extremities, providing critical insights into their demographic trends, clinicopathological hallmarks, immunohistochemical signatures, and clinical outcomes. Strengths of this review include the systematic aggregation of data and the detailed correlation of imaging and histopathology findings. However, the literature remains heavily reliant on isolated case reports and small series, thereby limiting the statistical power and external validity of these observations. Prospective or randomized controlled longitudinal studies are needed to clarify the true incidence, explore long-term recurrence rates more robustly, and investigate potential molecular targets for therapy. Multicenter registries focusing on rare vascular tumors of the extremities would be a logical next step, enabling higher-quality evidence on optimal management strategies, prognostic factors, and any links to concomitant fibroproliferative conditions such as Dupuytren’s disease [28,29]. Such collaborative efforts could ultimately refine both diagnostic accuracy and treatment algorithms, improving functional outcomes and quality of life for patients with these uncommon yet clinically significant tumors.

## 5. Conclusions

**Figure 7:**
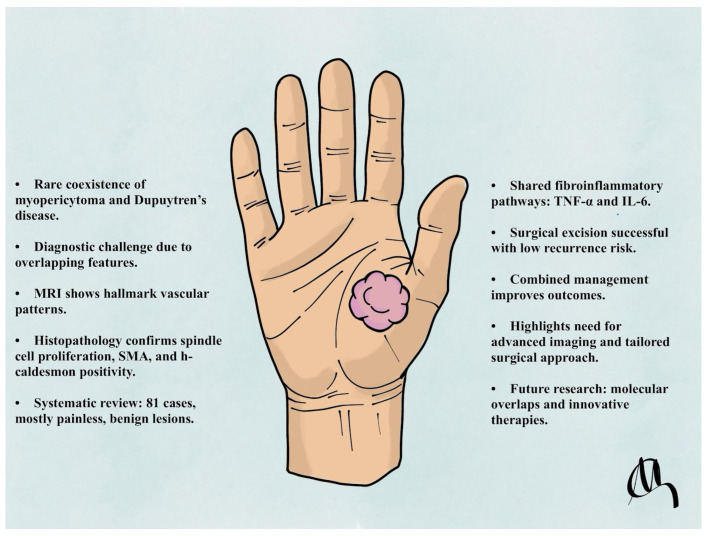
Schematic figure.

The coexistence of myopericytoma and DD highlights the complexities of diagnosing and managing rare overlapping pathologies. This case underscores the need for a multidisciplinary approach, combining advanced imaging, definitive histopathological analysis, and tailored surgical management to ensure accurate diagnosis and optimal outcomes. Identifying key molecular mechanisms, such as myofibroblast activation and inflammatory cytokine pathways, including tumour necrosis factor-alpha and interleukin-6, offers promising therapeutic advancements. Future research should focus on elucidating shared pathophysiological mechanisms, exploring targeted molecular therapies, and developing anti-fibrotic agents to improve treatment for recurrent or refractory cases. Collaborative efforts to document rare conditions and establish comprehensive registries could enhance diagnostic accuracy, refine management strategies, and address current knowledge gaps (Figure 7). 

## Figures and Tables

**Table 1:** Characteristics of included studies.

Case Report	Cases, *n*	Gender	Age, Years	Site	Clinical Presentation	Lesions	Size, mm	Histopathologic Features	Positive Immunohistochemistry	Local Recurrence (Follow-Up Period)
Mikami et al. [9]	1	Female	61	UE (hand)	AN	S	20	CPVP of ovoid spindle cells, vascular channels	SMA	Unknown
Mentzel et al. [10]	16	Mixed	13–87	UE	Unknown	S, multiple	Various	CPVP of spindle and plump myoid cells	SMA, h-caldesmon, desmin	Malignant in 1 (1 year), benign in others
Dray et al. [11]	5	Mixed	13–71	UE (hand)	AN	S, multiple	6–90	Bland round/oval cells, vascular channels	SMA	No recurrence (0.5–4 years)
Sadahira et al. [12]	1	Male	70	Volar aspect of left third, fourth and fifth digits	Non-painful	M, 3	5	Proliferation of spindle to oval shaped cells showing a concentric growth pattern	Positive for smooth muscle actin and vimentin	NS
Harreld et al. [13]	1	Female	36	Left palm mid diaphyseal region between index and long metacarpals	Sharp pain over left palm, no visible mass	S	5	Spindle cells with multiple small, scattered vascular spaces	Positive for smooth muscle actin	None (2 years)
Wagner and Shin [14]	1	Male	58	UE (hand)	Hypothenar mass, wrist pain	S	18	Spindle cells, perivascular growth	SMA, desmin	None (2 years)
Kara et al. [15]	1	Male	18	UE (hand)	Painful mass, ulnar nerve compression	S	24	Perivascular spindle-shaped cells	SMA, caldesmon	None (14 months)
Yang et al. [16]	1	Female	44	Ulnar aspect of the right distal forearm	Painless, slow-growing mass present for 5 years	S	40 × 20	Elongated blood vessels surrounded by spindle-shaped cells with bland morphology	Vimentin, smooth muscle actin, muscle-specific actin, h-caldesmon	None at 5 months
KF Seen [17]	1	Male	76	Right hand	Painless solitary nodule	S	45	Myoid appearing plump spindled cells with abundant eosinophilic cytoplasm	Positive for smooth muscle actin and calponin. Negative for S100 and CD34.	None
Jairajpuri et al. [18]	1	Male	52	UE (left thumb)	Painless nodule, 9 months duration	S	15 × 12	Proliferating spindle-to-ovoid bland cells with eosinophilic cytoplasm	SMA positive, negative for desmin and CD34	Unknown
Morzycki et al. [19]	1	Male	33	UE (left index finger)	Painless swelling, erythema	S	Not specified (extensive destruction of the distal phalanx)	Oval-to-spindle–shaped cells, eosinophilic cytoplasm, indistinct cellular border	SMA, vimentin	None (81 days)
Gulhane et al. [20]	1	Female	66	Hand (thumb)	Swelling, pain	S	24	CPVP of spindle cells, abundant myxoid stroma	SMA, vimentin	Unknown
Van Camp et al. [21]	1	Female	46	Base between the fourth and fifth fingers of the right hand	Non-painful, progressively enlarging mass over two years. Interfered with daily activities.	S	23 × 15 × 31	Well-defined tumour with spindle-shaped smooth muscle cells arranged around blood vessels. Diagnosis confirmed as a benign smooth-muscle neoplasm	Positive for alpha-smooth muscle actin (ASMA) and desmin. Negative for CD34, CD68, CD31, and Ki-67.	None (2 years)
Crombé et al. [22]	3	Mixed	Case 1: 72 Case 2: 30 Case 3: 51	Case 1: Ulnar palmar side of the hand Case 2: Palmar and ulnar side of the left wrist Case 3: Fifth finger of the right hand.	Case 1: Slowly growing lump over 5 years, progressively painful Case 2: Discrete lump with paraesthesia in the ulnar nerve territory Case 3: Slowly progressive lump, painless	S	Case 1: 54 Case 2: 45 Case 3: 9	Concentric perivascular growth pattern with fusiform cells, no atypia, low Ki-67 proliferative activity, strong staining for alpha-smooth muscle actin, desmin, and h-caldesmon.	Positive for ASMA, h-caldesmon, and desmin. CD34 was variably positive.	Case 1: No recurrence after 3 years Case 2: No recurrence after 9 years Case 3: No recurrence after 7 years
Kumar et al. [23]	1	Male	83	UE (right index finger)	Insidious, painless mass for 40 years, became painful after recent trauma. Firm, non-compressible, mobile, with overlying hyperpigmentation.	S	Not specified	Encapsulated soft tissue lesion with abundant vascular structures, proliferating spindle cells (myopericytes) with concentric perivascular growth. No nuclear atypia or abnormal mitotic activity.	Immunohistochemistry was not performed due to financial constraints	None (3 years)
Alhujayri et al. [24]	1	Male	45	Hand (1st webspace)	Fast-growing mass	S	30	Spindle cells, concentric vascular growth	SMA, caldesmon	None (6 months)
Ahatov et al. [25]	1	Female	45	Volar aspect of the left index finger middle phalanx	Painless slow growing mass	S	5	Concentric perivascular spindle cells	Positive for smooth muscle actin	None in 6 months
Menozzi et al. [26]	1	Male	54	Volar aspect of right thumb distal phalanx	Painful, solid, slow growing mass	S	NS	Ovoid spindle cells with elongated nuclei and eosinophilic cytoplasm	positive for smooth muscle actin stain and focal positivity for desmin	None (14 months)
Sullivan et al. [2]	1	Male	60	UE (hand)	Painful mass, gradual growth over 20 years	S	18 × 12 × 21	Non-encapsulated lesion with multiple blood vessels and concentric perivascular proliferation of spindle-shaped myoid cells. No nuclear atypia.	Positive for smooth muscle actin (SMA) and h-caldesmon. Negative for desmin and CD34	None
Awosusi et al. [27]	1	Male	29	Volar aspect of the right middle finger.	Painless swelling initially suspected to be an epidermal inclusion cyst.	S	15 × 15	Circumscribed, nodular soft tissue mass confined to the dermis; diagnosed as myopericytoma upon histopathologic evaluation	Positive for smooth muscle actin (SMA), h-caldesmon, and vimentin. Negative for CD34, CD68, S100, and desmin	None (1 year)

Abbreviations: AN: asymptomatic nodule; CPVP: concentric perivascular proliferation; H&N: head and neck; LE: lower extremities; PN: painful nodule; SMA: smooth muscle actin; UE: upper extremities; S: single lesion; M: multiple lesions.

**Table 2:** Quality Assessment.

	Selection	Ascertainment		Causality				Reporting	
Case Report	1	2	3	4	5	6	7	8	Overall Risk of Bias
Mikami et al. [9]	N	Y	U	Y	N/A	N/A	Y	Y	Low risk
Mentzel et al. [10]	N	U	Y	Y	N/A	N/A	N	Y	Moderate risk
Dray et al. [11]	U	Y	U	Y	N/A	N/A	U	Y	Low risk
Sadahira et al. [12]	N	U	Y	Y	U	N/A	N	Y	Moderate risk
Harreld et al. [13]	U	Y	Y	Y	N/A	N/A	Y	Y	Low risk
Wagner and Shin [14]	U	Y	Y	Y	N/A	N/A	U	Y	Moderate risk
Kara et al. [15]	U	Y	Y	Y	N/A	N/A	Y	Y	Low risk
Yang et al. [16]	U	Y	Y	Y	N/A	N/A	Y	Y	Low risk
KF Seen. [17]	N	U	Y	Y	U	N/A	N	Y	Moderate risk
Jairajpuri et al. [18]	U	Y	Y	Y	N/A	N/A	U	Y	Low risk
Morzycki et al. [19]	U	Y	Y	Y	N/A	N/A	U	Y	Low risk
Gulhane et al. [20]	U	Y	Y	Y	N/A	N/A	U	Y	Low risk
Van Camp et al. [21]	N	U	Y	Y	U	N/A	N	Y	Moderate risk
Crombé et al. [22]	U	Y	Y	Y	N/A	N/A	Y	Y	Low risk
Kumar et al. [23]	U	Y	Y	Y	N/A	N/A	Y	Y	Low risk
Alhujayri et al. [24]	U	Y	Y	Y	N/A	N/A	U	Y	Low risk
Ahatov et al. [25]	U	Y	Y	Y	N/A	N/A	U	Y	Low risk
Menozzi et al. [26]	U	Y	Y	Y	N/A	N/A	Y	Y	Low risk
Sullivan et al. [2]	U	Y	Y	Y	N/A	N/A	U	Y	Low risk
Awosusi et al. [27]	U	Y	Y	Y	N/A	N/A	Y	Y	Low risk

## Data Availability

No new data were created or analyzed in this study.

## Abbreviations

The following abbreviations are used in this manuscript:

AN	Asymptomatic nodule
CPVP	Concentric perivascular proliferation
H&N	Head and neck
LE	Lower extremities
PN	Painful nodule
SMA	Smooth muscle actin
UE	Upper extremities
S	Single lesion
M	Multiple lesion
DD	Dupuytren’s Disease
